# A Density Functional Theory Study on the Effects of Silver Doping on the Properties and Flotation Behavior of Jamesonite

**DOI:** 10.3390/molecules30071424

**Published:** 2025-03-23

**Authors:** Huimin Chen, Xi Yang, Yuqiong Li, Jianhua Chen

**Affiliations:** 1 School of Resources, Environment and Materials, Guangxi University, Nanning 530004, China; huiminchen123@163.com (H.C.); yql@gxu.edu.cn (Y.L.); 2 School of Chemistry and Chemical Engineering, Guangxi University, Nanning 530004, China; yangcece509@126.com; 3 Guangxi Key Laboratory of Processing for Non-Ferrous Metal and Featured Materials, Guangxi University, Nanning 530004, China

**Keywords:** jamesonite, DFT, flotation, collector, coordination chemistry

## Abstract

Silver (Ag) is a precious and valuable metal, and it has many carrier minerals. Through LA-ICP-MS analysis, it was found that jamesonite not only contains lead (Pb) and antimony (Sb) as precious metals but also trace amounts of Ag. In practice, the flotation method is generally used to recover these metals. This paper employs density functional theory calculations to demonstrate that after Ag doping in jamesonite, the Ag atoms exist in the lattice channels of jamesonite, and they form strong covalent bonds with the S atoms, resulting in strong interactions. When Ag is doped in the channels, the adsorption of sodium diethyldithiocarbamate (DDTC) as a collector on the Ag-doped jamesonite surface is the strongest, while that of butyl xanthate is the weakest. The adsorption interactions on the Ag-doped jamesonite surface are also stronger than on pure jamesonite. Coordination chemistry studies reveal that Ag^+^ undergoes a transition from a d^10^ to a d^9^s^1^ electronic configuration when incorporated into jamesonite, which increases its reactivity by generating unpaired electrons available for π-backbonding with collector molecules. Furthermore, owing to the high polarizability of Ag, the presence of Ag atoms alters the electronic environment of the surrounding Pb atoms, which enhances the π-backbonding interactions between the adsorbate reagent molecules and the Ag active sites. The research results are of great significance for the efficient recovery of Ag-containing jamesonite and provide a reference for the study of the properties of Ag-doped minerals.

## 1. Introduction

Jamesonite is a complex sulfosalt mineral with the chemical formula FePb_4_Sb_6_S_14_, which exists in nature as needle-like crystal structures in hydrothermal veins [1]. The Dachang Sn polymetallic deposit in Guangxi is an important resource of jamesonite worldwide. Many researchers have studied the jamesonite in Dachang [2], Guangxi, and found that it contains a large amount of the precious metal silver (Ag) with high economic value, which can enhance the mineral value as an additional metal. However, the existing state of Ag in industrial jamesonite is still unknown. Therefore, research on the occurrence state of Ag in industrial jamesonite is of great significance for the recovery and utilization of Ag resources.

Silver (Ag) has been regarded as a precious and valuable metal since antiquity, and it has maintained a prominent status throughout history to the present day. Ag is commonly used in various craftsmanship, optoelectronic materials, chemical materials, and biological applications [3,4]. The globally confirmed silver reserves are approximately 500,000 tons (2021), which is relatively small. Currently, there are generally believed to be four states of existence for silver: independent silver minerals, isomorphic silver, ion-absorbed silver, and amorphous silver [5,6,7,8]. The main carrier minerals of silver include galena, sphalerite, chalcopyrite, pyrite, etc. In addition, researchers have found that in the jamesonite in Dachang, Guangxi, there are associated precious metals such as antimony (Sb), lead (Pb), zinc (Zn), and silver (Ag) [9,10,11], which are of high economic value. Therefore, research has been conducted on Ag-containing jamesonite. In order to better recover the silver mineral in jamesonite, this study focuses on the laser ablation inductively coupled plasma mass spectrometry (LA-ICP-MS) micro-area in situ trace element determination of the Dachang ore samples, investigating the occurrence of Ag in jamesonite.

Due to the complex mineralization processes during formation, the composition of minerals undergoes corresponding changes. Studies have shown that when certain impurity elements are incorporated into the material structure, its properties can change significantly [1,2,12,13,14]. Zhou [15] studied the changes in the crystal structure and electronic properties of jamesonite after Sb and Pb replaced Fe at adsorption sites. The results showed that the structure and properties of jamesonite changed significantly after different amounts of Sb and Pb were substituted. With limited research on jamesonite, studies on galena and other minerals with similar properties are of great reference significance [16]. Muhammad [17] found that by doping varying concentrations of Ag into galena using hydrothermal techniques, the crystal size, unit cell volume, and porosity (%) of galena (PbS) increase with increasing Ag ion concentration. The presence of silver leads to changes in mineral crystal properties. Chen’s [18,19] research using density functional theory on Ag-containing galena revealed that the presence of Ag impurities enhances the interaction between galena and oxygen molecules and xanthate, indicating that Ag impurities promote the flotation of galena.

For minerals with complex properties like jamesonite, flotation is its most commonly used separation method [20,21]. Due to the presence of lead and antimony, two important metallic elements, jamesonite exhibits flotation characteristics similar to galena and stibnite in different separation environments. Collectors for jamesonite include xanthates, dithiophosphate, sodium diethyldithiocarbamate (DDTC) [22], aryl thiolate compounds [23], and others. Zhang et al. [3] used DDTC as a collector to study its flotation behavior on jamesonite, showing good floatability of jamesonite in the pH range of 2 to 13. Zhao et al. [24] conducted flotation experiments on jamesonite from Dachang using ethyl xanthate, finding similar flotation behavior to stibnite and good floatability at pH < 6. Cui [25] studied the flotation performance of jamesonite using a combination of 3418A and DDTC as collectors, and the combination significantly enhanced the flotation performance of jamesonite through physicochemical analysis. Yao [26] discovered that metal ion activation on the surface of sphalerite can significantly increase its activity, suggesting that Ag ions can change the d^10^ configuration on the surface of sphalerite to d^9^s^1^ when doped into the mineral.

Laser ablation (LA) combined with ICP-MS enables rapid qualitative and semi-quantitative analysis, providing spatial distribution information [27]. It is an accurate method for total quantitative analysis of trace elements, capable of precisely analyzing the distribution of Ag in jamesonite. Density functional theory, a method of quantum mechanics research, is widely applied in the computational and research study of solid-state physics. In this study, density functional theory (DFT) was used to investigate the presence of Ag in jamesonite and to explore the adsorption of common collectors (xanthates, dithiophosphate, and DDTC) on the surface of Ag-doped jamesonite. This study investigated the impact of Ag doping into jamesonite crystals on the flotation behavior of jamesonite and validated the flotation performance of the three collectors on Ag-doped jamesonite through micro-flotation experiments. The research findings are of significant theoretical and practical importance for the recovery of lead and antimony.

## 2. Results and Discussion

### 2.1. LA-ICP-MS Micro-Area In Situ Trace Element Determination

Currently, it is generally believed that Ag exists in four main forms: independent silver minerals, isomorphic silver, ion-absorbed silver, and amorphous silver [4,5]. At the microscopic scale, the existence of silver is categorized as visible silver (>1 μm) and invisible silver (<1 μm), where the invisible silver’s states mainly include lattice silver and submicroscopic inclusion silver [6].

Due to the limited literature on jamesonite, to confirm the presence of Ag in jamesonite, we conducted a trace element analysis using LA-ICP-MS on ore samples including jamesonite from Dachang. The LA-ICP-MS results showed that Ag can exist in jamesonite from Dachang Sn polymetallic deposits, with a distribution rate of Ag in jamesonite reaching 4.91%, existing as an impurity. The National Geological Experimental Testing Center conducted an LA-ICP-MS trace element analysis on pure jamesonite, as shown in Table 1. The table indicates the presence of Ag in various analysis sites within jamesonite, with an average silver content of 79.03 × 10^−6^ in jamesonite.

### 2.2. Existence States of Silver in the Lattice of Jamesonite

Quantitative analysis through LA-ICP-MS confirmed that jamesonite contains trace elements of Ag. To further investigate the state of Ag in jamesonite, the ore was expanded in the Z-axis direction and doped with Ag atoms through substitution and vacancy doping in simulation calculations.

#### 2.2.1. Lattice Substitution of Ag

Various potential substitution modes of Ag atoms in jamesonite exist. The formation energies and optimized crystal parameters after substitution are shown in Table 2.

**Figure 1:**
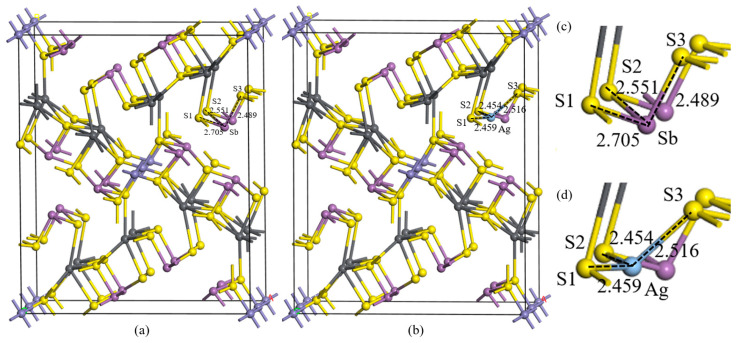
Unit cell structures of Ag substitution. Jamesonite (**a**), Ag substitution (**b**), jamesonite microstructure (**c**), and Ag substitution microstructure (**d**).

The substitution of Ag at 3-coordinate Sb sites exhibits the lowest formation energy (0.83 eV), making it the most feasible substitution pathway among all potential modes. However, the thermodynamic analysis reveals that Ag substitution in jamesonite remains energetically unfavorable, indicating a low likelihood of Ag existing in substitutional configurations. Following the substitution of Sb atoms, the crystal parameters of jamesonite were a = 15.925 Å, b = 19.404 Å, and c = 7.951 Å, slightly larger compared to the pure jamesonite crystal, but the deformation degree is smaller when Ag substitutes the 3-coordinate Sb atoms, indicating the most stable and easily occurring crystal deformation when Ag substitutes the 3-coordinate Sb atoms in jamesonite. The crystal structure of jamesonite will change at this point, as shown in Figure 1. Internal atomic distortions suggest that when Ag substitutes Sb atoms, the interaction between Ag and S atoms strengthens, leading to a reduction in bond lengths such as Sb–S1 from 2.705 Å to Ag–S1 at 2.459 Å; Sb–S2 from 2.551 Å to Ag–S2 at 2.454 Å; and Sb–S3 from 2.489 Å to Ag–S3 at 2.516 Å. With Sb’s atomic radius at 1.41 Å and Ag’s at 1.44 Å, it can be observed that Ag shows strong interactions with S, causing noticeable contractions in the Ag–S1 and Ag–S2 bond lengths, with an overall trend of contraction despite the increase in the Ag–S3 bond length.

#### 2.2.2. Vacancy Doping of Ag

**Figure 2:**
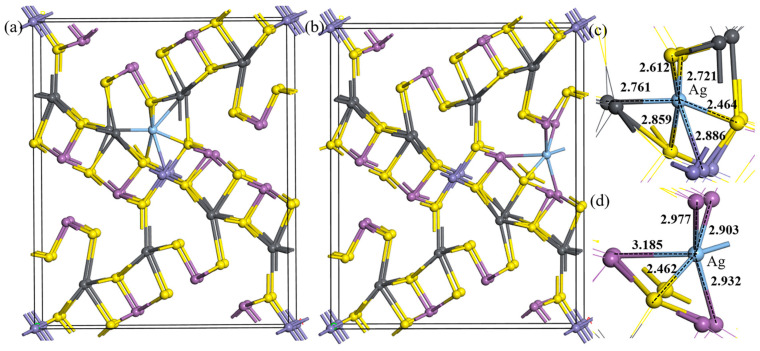
Unit cell structures of Ag vacancy doping. S–pb vacancy doping (**a**), S–Sb vacancy doping (**b**), S–pb vacancy doping microstructure (**c**), and S–Sb vacancy doping microstructure (**d**).

From the unit cell of jamesonite, it can be seen that the atoms in the crystal conform to the closest packing principle [28,29]. However, due to significant differences in atomic radii, they exhibit a branched distribution when connected at the microstructure level. According to the performance characteristics of the microstructure, this article categorizes the vacancy doping of jamesonite unit cells into two types: S–pb vacancy doping and S–Sb vacancy doping, as shown in Figure 2, where Figure 2a represents S–pb vacancy doping and Figure 2b represents S–Sb vacancy doping.

As shown in Table 3, when Ag atoms are vacancy-doped, the formation energies of the minerals are all <0, indicating that compared to substitution, under normal temperature and pressure, Ag is more likely to exist in jamesonite in the form of vacancy doping. When doping a single Ag atom, the formation energy of S–Sb vacancy doping is lower than that of S–pb vacancy doping, making S–Sb vacancy doping easier to form. The doped jamesonite unit cell exhibits significant deformation expansion. However, the deformation caused by S–Sb vacancy doping is noticeably smaller than that of S–pb vacancy doping. Moreover, compared to substitution doping, when doping a single Ag atom through S–Sb vacancy doping, the mineral unit cell shows an overall trend of contraction, with crystal parameters approaching those of pure jamesonite. Therefore, the Ag-doped jamesonite crystal cells obtained through S–Sb vacancy doping exhibit good stability.

When Ag atoms are doped into the interstitial spaces of the jamesonite, they form different interactions with other atoms. By Mulliken population analysis, the bonding interactions between atoms can be qualitatively determined. Table 4 shows the Mulliken population analysis of Ag atoms with other atoms in the lead crystal cell of jamesonite. It is observed that the Mulliken population analysis of the bonding between Ag and S atoms is all greater than 0.40, indicating a strong covalent interaction between Ag and S atoms. This suggests that Ag atoms readily form strong covalent bonds with S atoms, with bond lengths smaller than the sum of their atomic radii, indicating strong bond strength. For instance, the bonding length of Ag 1–S 29 is 2.46 Å, smaller than the sum of their atomic radii (Ag: 1.44 Å and S: 1.04 Å), with a Mulliken population value of 0.41, indicating strong covalent bonding. When Ag atoms are doped into jamesonite, interactions between Ag and Sb atoms are also present. The bonding length of Ag 1–Sb 7 is 2.90 Å, significantly larger than the sum of their atomic radii (Ag: 1.44 Å and Sb: 1.41 Å), with a Mulliken population of 0, indicating a weaker covalent interaction. The Table 4 shows that both the atomic radii of Ag and Sb are larger than the sum of their atomic radii, with Mulliken population values less than 0, highlighting the significantly weaker strength of interaction between Ag and Sb atoms. The Mulliken population analysis of the Ag−S bonds indicates that Ag atoms can stably exist in the unit cells of jamesonite and form strong covalent bonds with S atoms.

**Figure 3:**
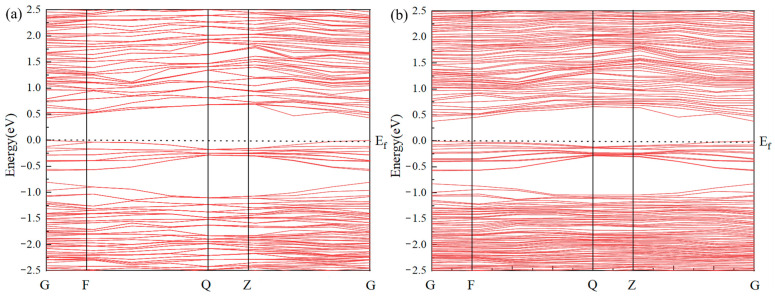
Band gap structure of jamesonite (**a**) and Ag-doped jamesonite (**b**).

The band structure of pure jamesonite and jamesonite doped with a single Ag atom through S–Sb vacancy doping is shown in Figure 3. This study reveals that the band gap of pure jamesonite is 0.431 eV [30], indicating a clear semiconductor structure. After Ag atom doping, the band gap of jamesonite is reduced to 0.379 eV. The Ag-induced impurity band introduces localized energy levels at 0.10 eV below the conduction band minimum (CBM) and 0.07 eV above the valence band maximum (VBM), narrowing the effective band gap. Additionally, partial overlap between the impurity band and the conduction band 0.05 eV above the Fermi level further reduces the energy barrier for electronic transitions. Overall, Ag doping into jamesonite modifies the band gap and the density of states near the Fermi level, significantly enhancing the electrical conductivity and surface reactivity of Ag-doped jamesonite. This promotes stronger π-backbonding interactions between collector molecules and Ag atoms, aligning with the observed improvements in flotation efficiency.

### 2.3. Adsorption Behavior of Ag-Doped Jamesonite Surface

#### 2.3.1. Jamesonite Surface Adsorption Site

**Figure 4:**
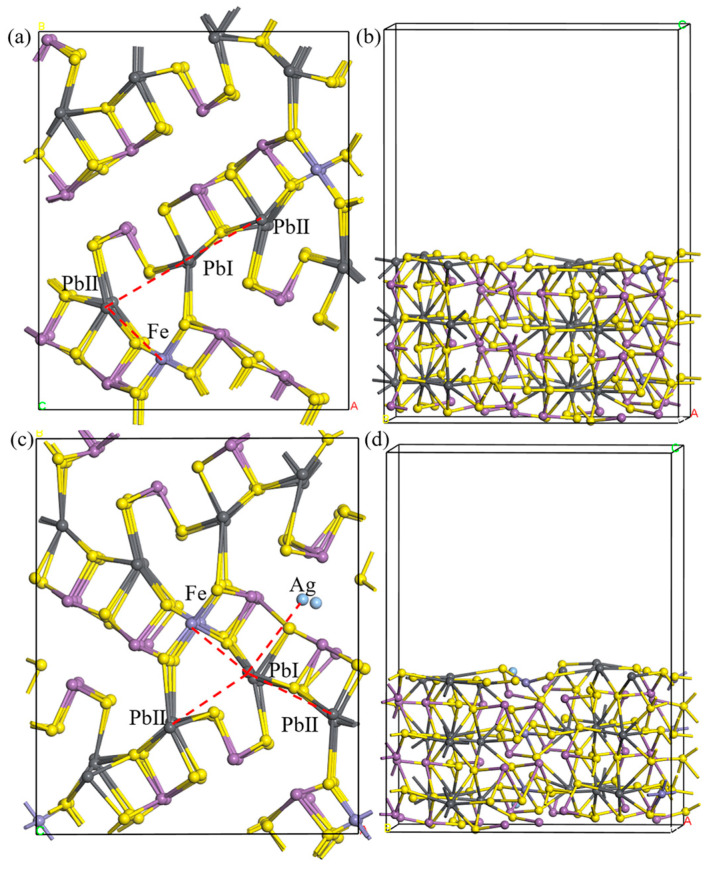
Jamesonite adsorption sites. Pure jamesonite (**a**,**b**), Ag-doped jamesonite (**c**,**d**).

The pure jamesonite (001) surface is shown in Figure 4a. Since the adsorption of collectors on the Pb-exposed surface of jamesonite (001) is the most stable within jamesonite [25], and Ag atoms are present in the channel-doped Pb-exposed surface of jamesonite (001), we chose the Pb-exposed surface of jamesonite as the research object. From Figure 4, it can be seen that the bond between Pb and nearby S atoms is relatively long, indicating a weaker bonding interaction, whereas Fe atoms are connected to S atoms in a covalent bond, indicating a stronger bonding interaction; Sb also forms relatively weak bonds with nearby S atoms, but stronger than the Pb–S bond. During the surface relaxation process, due to atomic deformations, the internal Fe atoms change from dsp^2^ hybridization (square planar) in the bulk phase to sp^2^ hybridization (trigonal planar), and based on the surface atomic positions, the adsorption sites for collectors on the jamesonite surface mainly include PbI–PbII vacancy adsorption, PbI–PbII bridging adsorption, and PbI–Fe bridging adsorption.

From Section 3.1, it can be seen that when Ag atoms undergo S–Sb vacancy doping in jamesonite, the lowest energy is formed. Therefore, we chose the jamesonite (001) surface doped with Ag through S–Sb vacancy doping for research, and the surface after doping is shown in Figure 4c. It can be observed from the figure that the surface of jamesonite doped with Ag atoms undergoes significant deformation, with displacements of Sb, Pb, and S atoms compared to the pure jamesonite surface.

The distances between the Ag atoms and nearby S atoms on the optimized Ag-doped jamesonite surface are around 2.4 Å, smaller than the sum of the atomic radii of Ag and S. This indicates that the Ag atom can stably exist on the surface of jamesonite. After Ag doping in jamesonite, the Pb–Ag sites are additionally present on the jamesonite (001) surface compared to the pure surface.

#### 2.3.2. Adsorption Structure of Collector on Jamesonite Surface

**Figure 5:**
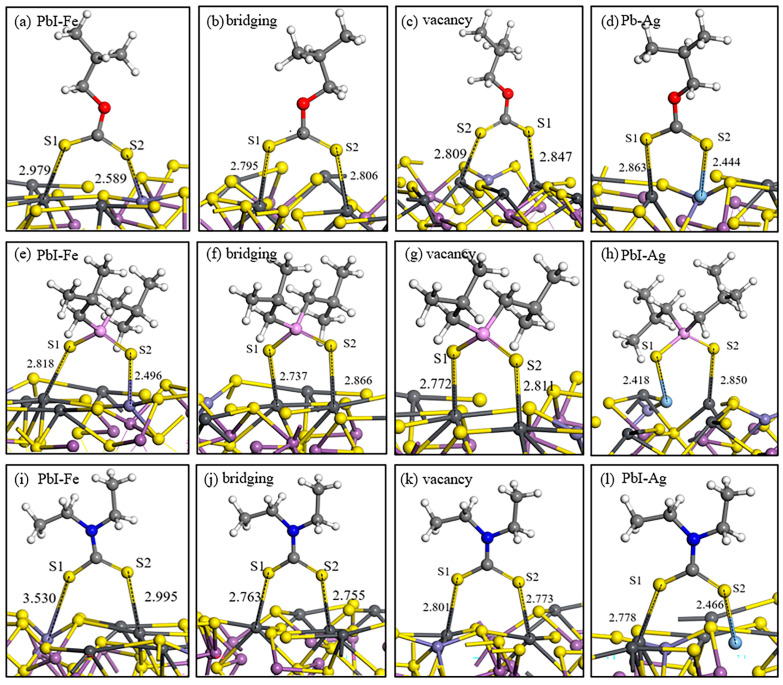
Adsorption structures of reagents on jamesonite surface. Butyl xanthate (**a**–**d**), 3418A (**e**–**h**), and DDTC (**i**–**l**).

The adsorption structures and energies of the three commonly used collectors on the surfaces of two types of jamesonite with different adsorption sites are shown in Figure 5 and Table 5. The strength of the interaction of butyl xanthate on the pure jamesonite surface adsorption sites is as follows: PbI–PbII bridging > PbI–PbII vacancy > PbI–Fe. The strongest adsorption occurs in the PbI–PbII bridging adsorption, where the Pb–S bond lengths are greater than the sum of the atomic radii (Pb: 1.75 Å and S: 1.04 Å), with the Pb–S1 bond being the strongest.

On the pure jamesonite surface, the strength of the 3418A adsorption sites is as follows: PbI–PbII vacancy > PbI–PbII bridging > PbI–Fe. The energy of the PbI–PbII vacancy adsorption is −232.37 kJ/mol, with a Pb–S1 bond length at 2.772 Å and a Pb–S2 bond length at 2.811 Å, indicating a strong interaction.

DDTC, as a commonly used collector for sulfide mineral flotation, exhibits strong selectivity. On the pure jamesonite surface, the strength of the interaction is as follows: PbI–PbII vacancy > PbI–PbII bridging > PbI–Fe. DDTC shows stronger interactions at different adsorption sites on jamesonite compared to butyl xanthate and 3418A, indicating the strongest effect on the pure jamesonite surface.

By comparing the adsorption of the three collectors on the pure jamesonite surface, it is evident that the effect on the doped jamesonite surface is significantly enhanced. Butyl xanthate has an adsorption energy of −211.06 kJ/mol on Pb–Ag sites while 3418A has an adsorption energy of −241.42 kJ/mol on Pb–Ag sites, and DDTC has an adsorption energy of −256.67 kJ/mol. The adsorption energies of the three collectors are higher than those on the pure jamesonite surface, with DDTC having the lowest adsorption energy on the Ag-doped jamesonite surface and the shortest Pb–Ag bond length. From the data, it can be concluded that the adsorption of collector molecules on the Ag sites of jamesonite is strongest, and DDTC shows the best effect, indicating that the presence of Ag atoms will benefit the flotation of jamesonite.

Comparing the adsorption effects of butyl xanthate, 3418A, and DDTC on the surface of jamesonite, it is evident that DDTC > 3418A > butyl xanthate, therefore indicating that DDTC has the best flotation effect. Through research on the adsorption of three collectors on the surface of Ag-doped jamesonite, it was found that the adsorption of collector molecules on the Pb–Ag sites is the strongest, indicating that Ag-doped jamesonite will promote a better interaction with collector molecules and achieve a more favorable flotation effect.

#### 2.3.3. Density of States of Collector Adsorption on Jamesonite Surface

**Figure 6:**
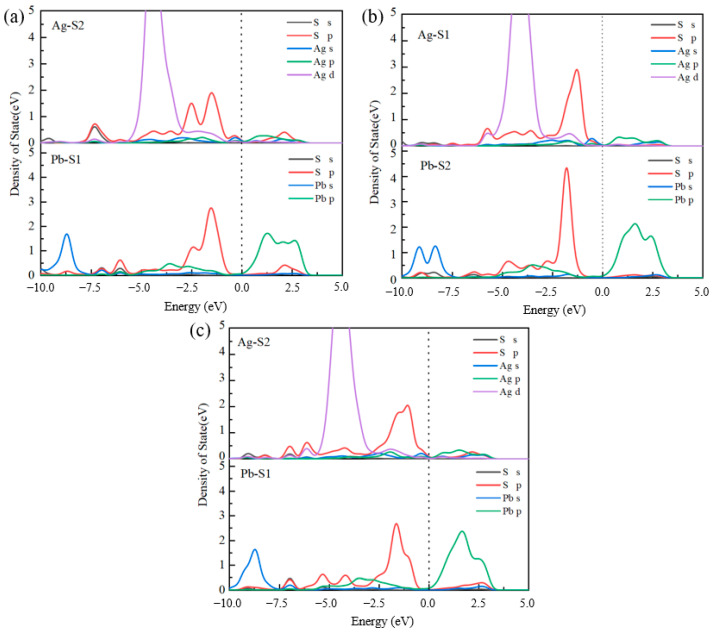
DOS of collector adsorption at Pb–Ag site. Butyl xanthate (**a**), 3418A (**b**), and DDTC (**c**).

From the adsorption structures of the three collectors on the mineral surface, it can be seen that the adsorption energy is lowest at the Pb–Ag adsorption sites on the surface of the doped jamesonite, with shorter bond lengths. Therefore, the density of states (DOS) for the adsorption of the three collectors at the Pb–Ag sites is as shown in Figure 6.

When butyl xanthate adsorbs at the Pb–Ag sites, resonance peaks primarily form between the Ag 4d orbitals and S 3p orbitals within the energy range of −6 to −4 eV. These peaks exhibit strong localization, indicating weak orbital hybridization interactions. Butyl xanthate has weaker adsorption at the Pb sites, with bonding interactions mainly occurring to the right of the Fermi level, showing mainly antibonding interactions in the lower energy range. When 3418A adsorbs on Pb–Ag sites, the 4d orbitals of Ag atoms and the 3p orbitals of S atoms form resonance peaks in the energy ranges of −6.5 to −5.5 eV and −5.5 to −4.5 eV, respectively. The resonance peak formed between Pb and S2 is mainly due to the 6p orbital of Pb and the 3p orbital of S, but the span of the resonance-formed DOS peak is significantly smaller than that of Ag atoms. The DOS of Ag atoms is more delocalized, with higher peaks in the −5.5 to −2.5 eV range. It is believed that 3418A has the strongest bonding strength at the Ag–S1 site when adsorbed on Pb–Ag sites.

In the case of DDTC adsorbing on the surface of jamesonite, the Ag–S2 bond formed shows the overlap of the 4d orbital of Ag and the 3p orbital of S atoms in the energy range of −7.5 to −2.5 eV, with many orbital hybridization peaks. The Ag–S bond hybridization peaks of DDTC are more delocalized compared to butyl xanthate and 3418A, resulting in stronger covalent interactions. Therefore, DDTC has the highest adsorption strength on the surface of Ag-doped jamesonite.

#### 2.3.4. Mulliken Charge Transfer at Ag Site

By analyzing the Mulliken charges of the collector molecules adsorbed on the surface of Ag-doped jamesonite, the nature of the interaction between the collector molecules and the mineral surface can be determined. The changes in Mulliken charges before and after the adsorption of the collector molecules are shown in Table 6. It can be observed from the table that Ag atoms on the surface of jamesonite lose electrons, while S atoms in the collector molecules gain electrons. It is believed that the main interaction produced by the collector molecules on the Ag atomic sites is covalent π-backbonding, which is relatively strong due to the loss of electrons from metal atoms and the gain of electrons by non-metal atoms. When butyl xanthate adsorbs on the Pb–Ag sites, Pb atoms gain electrons, resulting in a bonding interaction primarily driven by the ionic charge of Pb and S atoms, which is weaker. Both Ag and Pb atoms in 3418A and DDTC lose electrons, while S atoms gain electrons, resulting in stronger covalent interactions. The change in metal atom charge in DDTC is 0.17e, the largest change, indicating that DDTC produces the strongest interactions on the surface of Ag-doped jamesonite, corresponding to the adsorption results of the collector molecules.

### 2.4. Micro-Flotation Experiment Results

**Figure 7:**
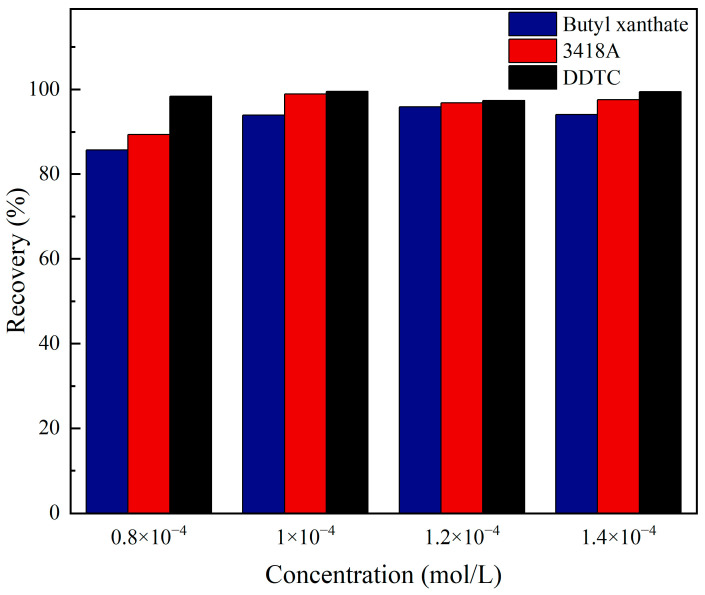
Micro-flotation experiment results of jamesonite recovered by different collectors.

The effects of different types of collectors on the flotation of jamesonite were studied through micro-flotation experiments. The micro-flotation experiment results are shown in Figure 7. It is evident from the figure that as the dosage of the three collectors increases to 1 × 10^−4^ mol/L, the recovery of jamesonite stabilizes at over 90%. Specifically, the recovery of jamesonite is 94.09% when butyl xanthate is used as the collector, 97.6% when 3418A is used as the collector, and 99.48% when DDTC is used as the collector. Under different dosages of collectors, DDTC consistently exhibits the strongest collecting performance, while butyl xanthate shows the weakest performance. Therefore, the recovery of the three collectors is in the following order: DDTC > 3418A > butyl xanthate, which is consistent with the simulated interaction results of the collectors on the surface of jamesonite.

### 2.5. Coordination Chemistry Principles of Ag-Doped Jamesonite

Research by scholars [26,31] has shown that when Ag atoms are doped into sphalerite, the d^10^ electron configuration of Ag^+^ transforms into a d^9^s^1^ configuration, resulting in four active π electrons in Ag^+^, which are highly active and can interact with the empty orbitals of collector molecules to form more stable π-backbonding, creating stronger covalent bonds.

Through the analysis of the charge transfer of collector molecules on the surface of Ag-doped jamesonite, it was found that strong covalent π-backbonding is formed between the collector molecules and Ag atoms, and the adsorption of the three collectors at the Pb–Ag sites is strongest. Therefore, it is believed that the presence of Ag atoms significantly enhances the adsorption of collector molecules on the surface of jamesonite. Based on the Mulliken charge transfer before and after adsorption of collectors, it is evident that DDTC forms relatively strong π-backbonding on the surface of Ag-doped jamesonite. Hence, it can be concluded that when Ag atoms are doped into jamesonite, Ag^+^ interacts more strongly with collector molecules. In addition, the polarizability of Ag^+^ is 1.72 Å^3^, while that of Pb is 0.615 Å^3^. The high polarizability of Ag^+^ also affects the surrounding Pb atoms, enhancing the adsorption of collector molecules on Pb atoms. Therefore, according to coordination chemistry principles, the effect of collectors adsorbing on the Ag sites of Ag-doped jamesonite is optimal.

## 3. Materials and Methods

### 3.1. LA-ICP-MS

The samples for testing were from the Dachang Sn polymetallic deposit in Guangxi, which were selected from the grinding products of the beneficiation plant at Dachang. Samples were collected daily from the grinding machine for a continuous week, mixed and fractionated to obtain a comprehensive ore sample, which is representative. The presence of Ag elements in the Dachang Mine samples and the analysis of trace elements in jamesonite were determined using laser ablation inductively coupled plasma mass spectrometry (LA-ICP-MS) by the Beijing General Research Institute of Mining and Metallurgy. LA-ICP-MS is a technique for laser ablation inductively coupled plasma mass spectrometry analysis, which can be used to quickly analyze the distribution and concentration of elements in samples. In the experiment, a laser beam was scanned across the sample surface to ablate particles, and the ablated particles were then analyzed by inductively coupled plasma mass spectrometry. LA-ICP-MS enables fast, high-throughput sample analysis, suitable for various sample types (such as rocks, minerals, biological tissues, etc.), capable of analyzing any element with high sensitivity, resolution, and repeatability. It can analyze the distribution and concentration of compounds at both micro and macro levels [27].

### 3.2. DFT Calculation Method

#### 3.2.1. Calculation Method

The jamesonite was computationally studied using the CASTEP module in Materials Studio software, with the PW91 functional generalized gradient approximation (GGA) used as the exchange-correlation functional [15]. To obtain more accurate computational parameters, the results of the cutoff energy calculations for the crystal cell of jamesonite are shown in Table 7. According to the calculation results, the optimal cutoff energy is determined to be 340 eV. Ultrasoft pseudopotentials were employed to describe the interaction between ions and valence electrons, with the selected valence electrons being Pb 6s^2^ 6p^2^, S 3s^2^3p^4^, Fe 3d^6^4s^2^, Sb 5s^2^5p^3^, and Ag 4d^10^5s^1^. The unit cell optimization algorithm used was BFGS. Self-consistent field (SCF) convergence was set to 2.0 × 10^−6^ eV/atom. The maximum displacement was 0.002 Å, and the maximum force was 0.05 eV/Å. The maximum energy was 2 × 10^−5^ eV/atom, and the maximum stress was 0.1 GPa. These parameters were used for the structural optimization of jamesonite, resulting in calculated lattice constants of a = 15.89 Å, b = 19.36 Å, and c = 3.96 Å, with a calculation error within 3%, meeting the computational requirements within experimental error limits. During the adsorption process, hydrogen ions were used to balance the charge, ensuring that the total system charge remained at 0.

#### 3.2.2. Calculation Model

**Figure 8:**
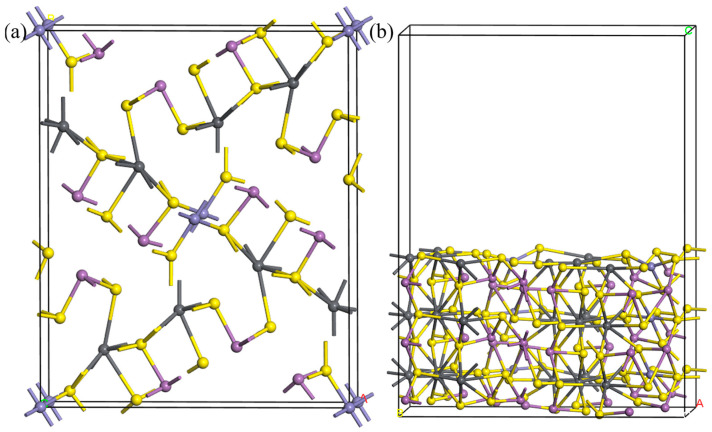
Calculation models of jamesonite cell (**a**) and jamesonite (001) surface (**b**).

The crystal structure of jamesonite is monoclinic [28], with the space group being P21/a. The experimental lattice parameters are a = 15.57 Å, b = 18.98 Å, and c = 4.03 Å [29]. The jamesonite crystal cell used in the calculations is shown in Figure 8. From the figure, it can be observed that the atomic coordination structure in the jamesonite crystal is quite complex. Each Pb atom forms an octahedral structure with six S atoms; Sb atoms form either three-coordinate trigonal pyramid structures or four-coordinate structures with S atoms; and Fe atoms form a four-coordinate planar square structure with four S atoms. In this study, the Ag atom was introduced by doping or substituting different positions of atoms to investigate the state of Ag in jamesonite. The relatively stable (001) cleavage plane of jamesonite was selected to study the adsorption of reagents on the surface of Ag-doped jamesonite.

#### 3.2.3. Formation Energy Calculation Method

The formation energy of doped atoms at different positions is calculated using the following formula:

(1) $$E_{b}=E_{\left(doped\;jamesonite\right)}-E_{\left(pure\;jamesonite\right)}-\mu_{\left(A\right)}+\mu_{\left(B\right)}$$

where *E_b_* represents the formation energy; *E*_(*doped jamesonite*)_ is the energy after Ag replaces the atoms in jamesonite; *E*_(*pure jamesonite*)_ is the energy of the original jamesonite; *μ*_(*A*)_ is the energy of the Ag atom; and *μ*_(*B*)_ represents the energy of the replaced atom.

The formula for calculating the adsorption energy [32,33,34,35] of reagents on the mineral surface is as follows:

(2) $$∆E_{ads}=E_{total}-E_{reagents}-E_{surface}$$

where *E_ads_* is the adsorption energy after the reagent molecules are adsorbed; *E_total_* is the total energy of the system after the reagent molecules are adsorbed on the surface; and *E_reagents_* and *E_surface_* are the total energies of the reagent molecules before adsorption and the surface, respectively. Lower adsorption energy indicates more stable adsorption of reagents on the mineral surface, while higher energy indicates decreased stability.

### 3.3. Micro-Flotation Experiment

**Figure 9:**
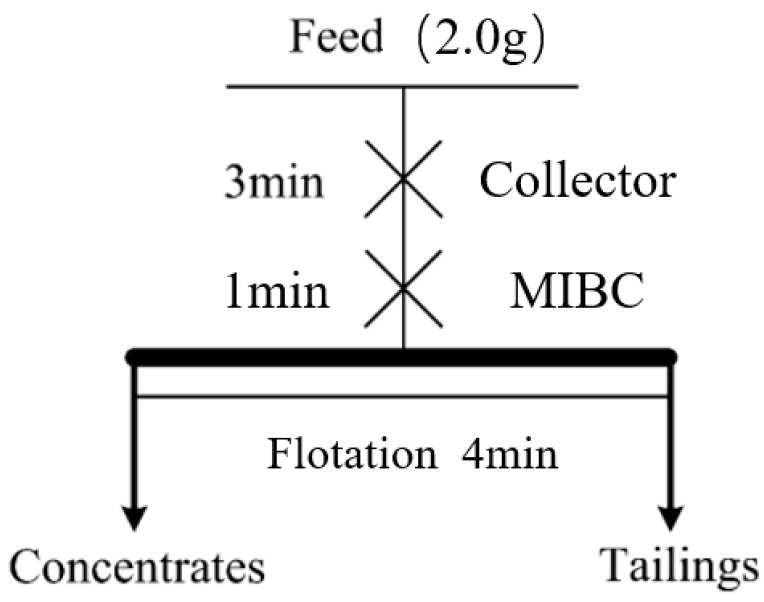
Flowsheet of micro-flotation experiment.

A micro-flotation experiment [36,37,38,39,40,41,42] used jamesonite from Dachang in Guangxi as the research subject. Under pH = 7 conditions, the study explored the effect of the dosage of three different collectors on the recovery of Ag-containing jamesonite. The research was conducted using an XFGCII.5 laboratory inflatable hanging groove flotation machine for the pure mineral flotation experiment. The experimental procedure is illustrated in Figure 9, and the results are shown in Figure 2.

## 4. Conclusions

- An LA-ICP-MS analysis determined that Ag existed in the lattice of jamesonite from Dachang. Using density functional theory, it was found that Ag existed in the form of lattice S−Sb vacancy doping in jamesonite. A Mulliken population and band structure analysis revealed that the doped Ag atoms could form strong covalent bonds with surrounding S atoms, resulting in strong interactions.
- An analysis of the adsorption structures of the three different collectors on the surface of jamesonite indicated that the doping of Ag strengthened the adsorption of collectors on the surface of jamesonite. The adsorption of the three collectors at the Pb–Ag sites was strongest, with the following order of adsorption strength: DDTC > 3418A > butyl xanthate.
- Micro-flotation experiments on jamesonite from Dachang, Guangxi, showed that all three collectors exhibited strong collecting abilities. Under different dosages, the collecting performance of the three collectors for jamesonite was in the following order: DDTC > 3418A > butyl xanthate.
- Studies on the coordination chemistry principles of Ag atom doping revealed that when Ag atoms were present in jamesonite, the d^10^ electron configuration of Ag^+^ transforms into a d^9^s^1^ configuration, leading to increased activity. Due to the large polarizability of Ag, it affects the surrounding Pb atoms, enhancing the formation of π-backbonding by collector molecules at Ag sites.

## Figures and Tables

**Table 1:** LA-ICP-MS analysis of trace elements.

Ore Type	Point Number	LA-ICP-MS Analysis of Trace Elements (ω_B_/10^−6^)								
		Mn	Cu	Zn	As	Ag	Ba	In	Sn	Zr
Jamesonite	JMT–1	526.45	8.09	124.71	381.66	6.74	2.2	288.66	1420.82	0.11
	JMT–2	342.78	8.99	714.47	121.59	29.28	12.12	264.69	518.25	0.03
	JMT–3	794.77	3.58	256.46	101.96	5.56	1.12	197.52	934.67	–
	JMT–4	612.97	64.09	152.46	196.15	4.55	2.76	212.01	1726.19	286
	JMT–5	1104.14	1.22	79.33	711.26	10.93	0.88	227.87	2402.91	0.1
	JMT–6	953.93	35.44	5146.24	458.63	22.6	10.27	215.05	2063.65	–
	JMT–7	1213.55	187.02	4867.8	262.53	144.71	20.63	187.65	1616.08	0.82
	JMT–8	1115.27	2.86	85.99	105.47	10.19	4.56	240.39	829.12	0
	JMT–9	1011.65	50.69	177.44	246.31	601.27	2.03	171.65	3349.72	102.62
	JMT–10	859.24	9.91	97.91	418.89	91.28	1.82	182.64	1787.08	–
	JMT–11	1092.1	123.15	39.28	864.28	22.74	1.14	230.47	5674.65	–
	JMT–12	1093.55	5.92	1255.23	244.76	10.71	7.37	287.61	755.81	14.5
	JMT–13	154.73	1.37	109.61	186.51	2.82	7.75	194.15	803.87	0
	JMT–14	1121.33	9.16	373.84	791.03	211.87	2.26	138.37	4418.73	–
	JMT–15	1163.45	0.48	0	35.19	10.15	0.93	347.68	539.97	0.07
	JMT–16	877.33	34.13	898.72	341.75	79.03	5.19	225.76	1922.77	26.95

**Table 2:** Formation energy of different substitution sites.

Substitution Type	a	b	c	α	β	γ	Formation Energy/(eV)
Jamesonite	15.88	19.36	7.92	90.00	92.27	90.00	0
2–coordinate S	16.06	19.74	7.93	89.66	91.59	89.75	2.17
3–coordinate S	16.04	19.81	8.05	89.94	92.40	90.15	2.98
4–coordinate S	15.92	19.33	7.96	90.08	91.74	90.00	2.02
5–coordinate S	16.09	19.73	7.84	89.79	92.09	89.91	2.51
4–coordinate Fe	15.96	19.79	8.07	90.16	92.59	89.94	2.33
6–coordinate Pb	15.89	19.36	7.90	89.92	92.34	90.24	1.64
4–coordinate Sb	15.90	19.35	7.92	90.15	92.30	90.12	1.16
3–coordinate Sb	15.92	19.40	7.95	90.06	92.18	89.99	0.83

**Table 3:** Formation energy of different doping forms.

Doping Form	a	b	c	α	β	γ	Formation Energy/(eV)
Jamesonite	15.88	19.36	7.92	90.00	92.27	90.00	0
S–Pb vacancy doping	16.028	19.348	7.961	90.130	92.101	90.013	−0.668
S–Sb vacancy doping	15.923	19.534	7.980	90.011	92.355	89.865	−1.158

**Table 4:** Mulliken bond population of Ag S–Sb vacancy doping unit cell.

Bond	Population	Length (Å)
S 29–Ag 1	0.41	2.46
S 20–Ag 1	0.40	2.51
Ag 1–Sb 7	0.00	2.90
Ag 1–Sb 2	−0.03	2.93
Ag 1–Sb 19	−0.06	2.98

**Table 5:** Adsorption results of reagent molecules on jamesonite surface.

Mineral Type	Reagent	Adsorption Site	Adsorption Energy (kJ/mol)	M–S1 (Å)	M–S2 (Å)
Jamesonite	Butyl xanthate	PbI–Fe	−114.89	2.589	2.979
		PbI–PbII bridging	−161.98	2.795	2.806
		PbI–PbII vacancy	−156.32	2.847	2.809
	3418A	PbI–Fe	−119.33	2.818	2.496
		PbI–PbII bridging	−208.91	2.737	2.866
		PbI–PbII vacancy	−232.37	2.772	2.811
	DDTC	PbI–Fe	−174.59	3.53	2.995
		PbI–PbII bridging	−242.97	2.801	2.773
		PbI–PbII vacancy	−253.98	2.763	2.755
Ag-doped jamesonite	Butyl xanthate	Pb–Ag	−211.06	2.863	2.444
	3418A	Pb–Ag	−241.42	2.85	2.418
	DDTC	Pb–Ag	−256.67	2.778	2.466

**Table 6:** Mulliken charge transfer before and after adsorption of different collectors.

Reagent	Adsorption State	Mulliken Charge (e)			
		S1	S2	Ag	Pb
Butyl xanthate	Before	0.00	−0.1	−0.1	0.54
	After	−0.14	−0.12	−0.05	0.52
3418A	Before	−0.4	−0.37	−0.1	0.54
	After	−0.55	−0.51	−0.08	0.6
DDTC	Before	0.03	−0.02	−0.1	0.54
	After	−0.23	−0.15	−0.06	0.67

**Table 7:** Calculation results of cutoff energy.

Cutoff	Energy/(eV)	Lattice/(Å)			
		a	b	c	β
250	−24,641.30	16.23	19.52	4.11	92.10
280	−24,641.24	15.91	19.91	3.94	92.59
310	−24,641.74	15.84	19.32	3.95	92.23
340	−24,642.09	15.89	19.36	3.96	92.27
370	−24,641.75	15.85	19.62	3.93	91.77
400	−24,641.85	16.06	19.59	3.93	91.83
430	−24,641.5	15.82	19.42	3.91	91.58

## Data Availability

The data that support the findings ofthis study are available from thecorresponding author upon reasonable request.
